# COVID-19 severity and risk of SARS-CoV-2-associated asthma exacerbation by time since booster vaccination: a longitudinal analysis of data from the COVIDENCE UK study

**DOI:** 10.1136/bmjresp-2025-003158

**Published:** 2025-05-15

**Authors:** Giulia Vivaldi, Mohammad Talaei, Paul E Pfeffer, Seif O Shaheen, Adrian R Martineau

**Affiliations:** 1 Blizard Institute, Barts and The London School of Medicine and Dentistry, Queen Mary University of London, London, UK; 2 Wolfson Institute of Population Health, Barts and The London School of Medicine and Dentistry, Queen Mary University of London, London, UK; 3 Department of Respiratory Medicine, Barts Health NHS Trust, London, UK; 4 William Harvey Research Institute, Barts and The London School of Medicine and Dentistry, Queen Mary University of London, London, UK; 5 Allergy and Lung Health Unit, The University of Melbourne School of Population and Global Health, Melbourne, Victoria, Australia; 6 Asthma UK Centre for Applied Research, Queen Mary University of London, London, UK

**Keywords:** Asthma, COVID-19

## Abstract

**Background:**

COVID-19 booster vaccinations are offered annually to priority groups, but many people have not been vaccinated in over a year. We therefore assessed the association between time since booster vaccination and breakthrough infection characteristics. We also explored whether incident COVID-19 associates with asthma exacerbations in boosted individuals with asthma and if the risk of COVID-19-associated exacerbation is affected by time since vaccination.

**Methods:**

COVIDENCE UK is a prospective, longitudinal, population-based study of COVID-19. We included adult participants who had received ≥1 booster vaccination. Time since vaccination was binarised at 6 or 12 months according to vaccine eligibility subgroup. We used regression models to obtain adjusted estimates for the association between time since vaccination and breakthrough infection severity (requiring bedrest vs milder symptoms), symptom duration, and impact on health-related quality of life (EQ-5D-3L Index). We then assessed the association of incident COVID-19 with asthma exacerbations using multilevel mixed models, by time since vaccination.

**Results:**

7391 boosted participants reported at least one breakthrough infection. Across all eligibility subgroups, greater time since vaccination associated with increased odds of severe symptoms (ORs ranging from 1.31 (95% CI 1.06 to 1.62) to 1.61 (1.29 to 2.01)). Not receiving a booster vaccination in the previous 12 months was associated with longer time to recovery overall (HR for recovery 0.90, 95% CI 0.81 to 0.99), but evidence for vaccination subgroups was weak. Greater time since vaccination was associated with a small decrease in EQ-5D-3L Index overall (−0.02, 95% CI −0.03 to −0.00) and among participants younger than 75 years, but did not reach our estimates for a minimum clinically important difference. Among 2100 participants with asthma, incident COVID-‍19 associated with increased risk of asthma exacerbation, both within 12 months of vaccination (OR 5.11 (95% CI 4.19 to 6.24)) and later (5.60 (2.98 to 10.53)), with a greater difference in point estimates when considering severe exacerbations (6.59 (4.70 to 9.22) vs 9.20 (3.56 to 23.78)).

**Conclusion:**

Longer time since booster vaccination consistently associates with more severe infections and may increase the risk of severe asthma exacerbations in people with asthma. These findings highlight the importance of ensuring those currently eligible receive their boosters, and the need for research on further vaccinations in people with asthma no longer eligible for boosters.

**WHAT IS ALREADY KNOWN ON THIS TOPIC**

- Greater time since COVID-19 vaccination has been shown to associate with increased symptom severity of breakthrough infection, but follow-up has been limited and little is known about its impact on symptom duration or acute changes to health-related quality of life.
- Incident COVID-19 is known to trigger asthma exacerbations in people with asthma, but this relationship has not been examined in detail in a boosted population, nor by time since vaccination.

**WHAT THIS STUDY ADDS**

- In our study, greater time since vaccination associated with more severe symptoms when infected, but we observed little evidence of association with symptom duration and little impact on health-related quality of life.
- Incident COVID-19 continued to associate with increased risk of asthma exacerbations in boosted individuals with asthma, with evidence that greater time since vaccination may increase the risk of severe asthma exacerbations.

**HOW THIS STUDY MIGHT AFFECT RESEARCH, PRACTICE OR POLICY**

- Our findings highlight the importance of ensuring those currently eligible receive their booster vaccinations, and the need for research on further vaccinations in people with asthma no longer eligible for boosters.

## Introduction

 More than 3 years since the start of vaccination programmes against SARS-CoV-2, booster vaccinations continue to be offered in countries across the world.^1^ Within the context of Omicron dominance and high population immunity, the WHO has published recommendations of priority groups for repeated booster vaccinations;^2^ these recommendations are broadly followed by several countries,^3 4^ with others continuing to offer annual vaccinations for everyone.^5^

In the UK, booster vaccinations are offered annually to priority groups in the autumn,^6^ with more than 7 million people aged 65 years and older vaccinated in autumn 2023.^7^ Some more vulnerable groups, such as adults aged 75 years and older, care home residents, and the immunosuppressed, are additionally offered a vaccine in the spring.^6^ However, eligibility for SARS-CoV-2 booster vaccinations is continually under review,^4^ with the offering reduced each autumn to date.^6^

Vaccine effectiveness is known to wane over time;^8^ therefore, longer time since vaccination, either because of vaccine ineligibility or under-vaccination,^9^ may put people at increased risk of infection and severe disease. Vaccine protection against severe outcomes lasts longer than protection against mild disease;^10^ however, mild-to-moderate COVID-19 remains an important concern, with acute COVID-19 and long-term sequelae continuing to be a major reason for sickness absence in the workforce.^11^ In people with breakthrough SARS-CoV-2 infection, greater time since vaccination has been shown to associate with greater symptom severity^12 13^ and increased risk of severe or critical COVID-19.^14^,^16^ However, few studies have looked at symptom duration,^12^ and no studies, to our knowledge, have assessed how time since booster vaccination may affect the infection’s impact on health-related quality of life. Additionally, very few studies have considered outcomes more than 8 months after booster vaccinations,^14 17^ whereas an increasing portion of the UK population has not received a SARS-CoV-2 vaccine for a year or more.^6^

Among those currently eligible for annual booster vaccination are people with poorly controlled asthma.^6^ In this population, the protective effect of vaccination is twofold: on the one hand, vaccination protects against severe COVID-19, for which poorly controlled asthma is a risk factor;^18^ on the other, by reducing the incidence and severity of breakthrough infections, vaccination may also protect against asthma exacerbations triggered by incident COVID-19^19 20^ and subsequent worsening of asthma control.^21^ However, existing studies into the relationship between incident COVID-19 and asthma exacerbations have not been carried out in boosted populations.^19 20^ Additionally, these studies have not considered whether exacerbation risk from breakthrough SARS-CoV-2 infection is affected by time since vaccination, which is likely to be longer in people with well-controlled asthma, who may still experience severe asthma exacerbations^22^ but are currently not eligible for booster vaccinations.^6^

To address these knowledge gaps, we therefore aimed to assess the association between time since vaccination and various characteristics of breakthrough infections, including symptom severity, symptom duration, and acute changes to health-related quality of life. We also aimed to evaluate whether incident COVID-19 continued to be associated with asthma exacerbations in a boosted population with asthma, and whether this association was affected by time since booster vaccination.

## Methods

### Study design and participants

COVIDENCE UK is a prospective, longitudinal, population-based observational study of COVID-19 in the UK population (https://www.qmul.ac.uk/covidence).^23^ Inclusion criteria were age 16 years or older and UK residence at enrolment, with no exclusion criteria. Participants were invited via a national media campaign via print and online newspapers, radio, television, social media, and online advertising. Enrolled participants completed an online baseline questionnaire and monthly follow-up questionnaires to capture information on potential symptoms of COVID-19, results of nose or throat swab tests for SARS-CoV-2, vaccinations, and asthma exacerbations. The study was launched on 1 May 2020 and closed to enrolment on 6 October 2021. The final COVIDENCE UK cohort was majority female (70.2%) and white (93.7%), with under-representation of people younger than 50 years, men, and minority ethnicities.^23^ This study is registered with ClinicalTrials.gov, NCT04330599.

In this analysis, we included all participants who had received at least one COVID-19 booster vaccination and reported a positive nose or throat swab test for SARS-CoV-2 at least 14 days after their last vaccination. This analysis was pragmatic in nature, including all participants meeting these eligibility criteria with no sample size specified.

### SARS-CoV-2 infection

We defined evidence of a SARS-CoV-2 infection as a positive SARS-CoV-2 swab test. Infection dates were defined as the date of the positive test. Infections reported in the 14 days following a vaccination were excluded. Participants with symptoms were asked to report the severity of their symptoms as mild (able to do most of usual activities), moderate (unable to do usual activities, but without requiring bedrest), or feeling very unwell (requiring bedrest). Participants were asked to report their symptom onset date and whether they felt back to normal on the day of the questionnaire; if they responded yes, they were asked to report a symptom end date. Participants not reporting symptoms were classified as asymptomatic.

### Booster vaccine eligibility groups

**Figure 1:**
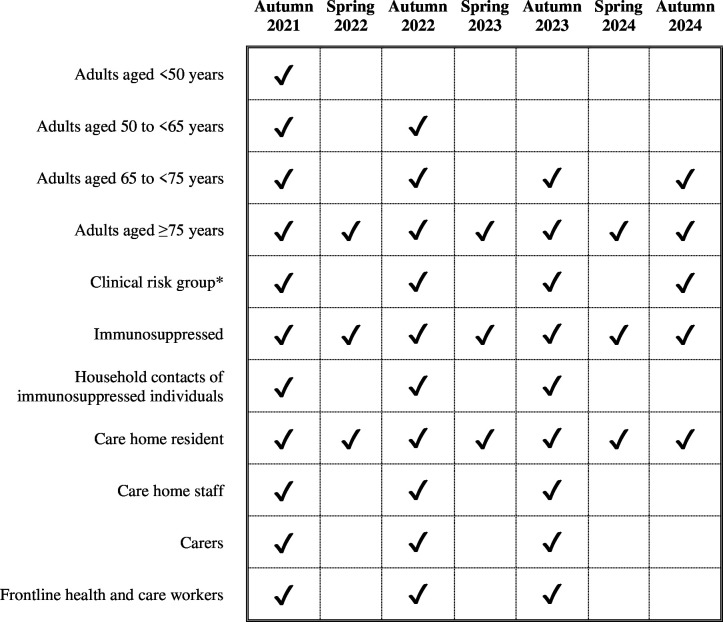
Vaccine eligibility. *The clinical risk group defined by the Joint Committee on Vaccination and Immunisation is comprised of individuals with a wide range of health conditions, outlined in online supplemental table S1.

After the emergence of Omicron, all adults in the UK were eligible for the first round of booster vaccinations, which began in autumn 2021; however, additional booster vaccinations have been restricted to specific subgroups according to their risk of severe outcomes, their contact with potentially vulnerable people, and to protect NHS capacity.^6^ The requirement for further vaccinations is regularly reviewed by the Joint Committee on Vaccination and Immunisation,^6^ leading to some groups no longer being eligible for further vaccinations (figure 1).

Our interest was in how time since booster vaccination affects infection characteristics. We therefore focused on eligibility groups designed to avoid severe outcomes in the individual (ie, related to age and clinical risk), rather than preventing onwards transmission (ie, regarding care homes, social and care staff, and household contacts of at-risk populations).

COVIDENCE UK does not have data on all of the eligibility criteria for the clinical risk group; we therefore classified participants as being in the clinical risk group if they reported chronic obstructive pulmonary disease or poorly controlled asthma (based on medications taken or exacerbations reported in monthly questionnaires; see online supplemental table S1), diabetes, heart disease, peripheral vascular disease, kidney disease, major neurological conditions, immunosuppression, morbid obesity, or splenectomy. We did not consider immunosuppressed individuals separately owing to incomplete data on immunosuppression (eg, people undergoing therapies that cause immunosuppression) and low numbers.

### Asthma exacerbations

Participants were asked to report asthma exacerbations occurring since their last questionnaire. Exacerbations were classified as severe if they required treatment with systemic corticosteroids or precipitated emergency department attendance or hospital admission.

### Time since booster vaccination

Time since booster vaccination was defined as the number of days between the last vaccine received and the infection date. To assess whether more frequent vaccination would be beneficial for particular subgroups, we converted time since vaccination into a binary variable, classifying participants as having received a vaccine in the past 6 months (for those currently eligible for annual vaccination, but not 6-monthly vaccination) or the past 12 months (for those currently not eligible for annual vaccination).

### Covariates

Potential confounders of the association between time since vaccination and breakthrough COVID-19 severity were identified using a directed acyclic graph (online supplemental figure S1). While many risk factors have been identified for greater COVID-19 severity after vaccination,^24^ we specifically considered those that might affect vaccine eligibility and vaccine uptake, which could both impact time since last vaccination. Owing to the aims of the UK vaccine programme, most of the factors that affect vaccine eligibility also affect disease severity.^6^ Vaccine uptake or hesitancy is associated with various factors not captured in COVIDENCE UK, such as compliance with health restrictions and engaging with conspiracy theories;^25^ however, it has also been found to associate with factors such as age, sex, general health, and socioeconomic status.^26^ We therefore adjusted for age and other vaccine eligibility criteria (ie, comorbidities and frontline worker status), sex, ethnicity, socioeconomic status (represented by level of educational attainment and Index of Multiple Deprivation), general health, previous SARS-CoV-2 infection, and the number of vaccines received at time of infection. We additionally adjusted for whether infection dates were before or after April 2022, when access to COVID-19 testing was greatly reduced,^27^ as this is likely to have affected the severity distribution of recorded infections.

### Statistical analysis

First, we assessed the potential impact of time since vaccination on three dimensions of SARS-CoV-2 infection: reported symptom severity, duration of symptoms, and health-related quality of life.

We used logistic regression to explore the association between time since vaccination and self-reported symptom severity; to do so, we converted symptom severity into a binary variable (very unwell vs asymptomatic, mild, or moderate symptoms). We compared differences in symptom duration using Cox regression, censoring participants at the questionnaire date if they reported not feeling back to normal but did not subsequently report a symptom end date for that infection episode. Finally, we examined changes in health-related quality of life using the EQ-5D-3L,^28^ which participants completed in each monthly questionnaire. We converted the five dimensions of the EQ-5D-3L into a weighted health state index, using EQ-5D preference weights obtained from the UK general population,^29^ and calculated the difference between the average value in the 6 months before infection and the value in the month immediately following the infection. We then assessed the association between time since vaccination and change in EQ-5D-3L index using linear regression. As some participants reported more than one SARS-CoV-2 infection, we used cluster-robust standard errors to account for repeated measures.

Second, we evaluated whether incident SARS-CoV-2 infection continued to be associated with asthma exacerbations in a boosted population with underlying asthma, and whether this association was affected by time since vaccination. We included all boosted participants with asthma and post-booster follow-up. We excluded any observations in the first month after a SARS-CoV-2 vaccination as we could not guarantee that any exacerbations reported would have occurred after the vaccination. We calculated ORs for associations between incident SARS-CoV-2 infection and both mild and severe asthma exacerbations using multilevel logistic generalised linear models, with random effects for participants. We included SARS-CoV-2 infection as a three-level independent variable: no infection, infection when vaccinated in the previous 12 months, and infection when vaccinated more than 12 months prior. We adjusted for the same covariates as in the SARS-CoV-2 infection models, while additionally adjusting for asthma treatment level (use of reliever inhaler only, use of inhaled corticosteroid, use of a long-acting bronchodilator inhaler, or monoclonal antibody therapy), asthma exacerbation history, incident non-COVID-19 acute respiratory infection, tobacco smoking status, season (October–April vs May–September), and measures of response rate and SARS-CoV-2 testing rate. Previous SARS-CoV-2 infection, incident non-COVID-19 acute respiratory infection, tobacco smoking status, and season were included as time-varying covariates in these multilevel models.

We did three sensitivity analyses. First, we restricted the analyses to participants with previous infection in order to control for the severity of their most recent infection. In doing so, we aimed to factor in participants’ predisposition towards severe infections, which may not be fully accounted for by the covariates available to us. Second, as the analyses are restricted to people who report positive test results, we are effectively conditioning on the likelihood of being infected and taking a SARS-CoV-2 test, putting us at risk of collider bias.^30^ We therefore carried out inverse probability-weighted regressions, with weights based on the probability of reporting a positive test calculated on all participants who had received a booster vaccination. Third, to evaluate the possibility of informative censoring in our Cox regression models, we relaxed the assumption of independent censoring using multiple imputation of censored symptom durations,^31^ varying the change in the hazard function (γ) after censoring for censored participants from −10 to 10, and imputing 100 datasets for each value of γ.

The proportional hazards assumption for the Cox models was tested using Schoenfeld residuals and visual assessment of log–log plots of survival; models were then stratified by variables found to violate the assumption to assess the impact on our estimates. Missing data were handled with listwise deletion or multiple imputation (sensitivity analysis alone). Analyses were done with Stata V.18.0 or R (V.4.4.2).

### Patient and public involvement

A team of patient and public involvement (PPI) representatives was put together at the COVIDENCE UK study inception, to guide the study design and development of participant-facing materials. Discussions with the PPI team led to revisions of the study questionnaire. Research priorities for COVIDENCE UK were also informed by study participants, whose feedback was requested through the study webinars.

## Results

**Figure 2:**
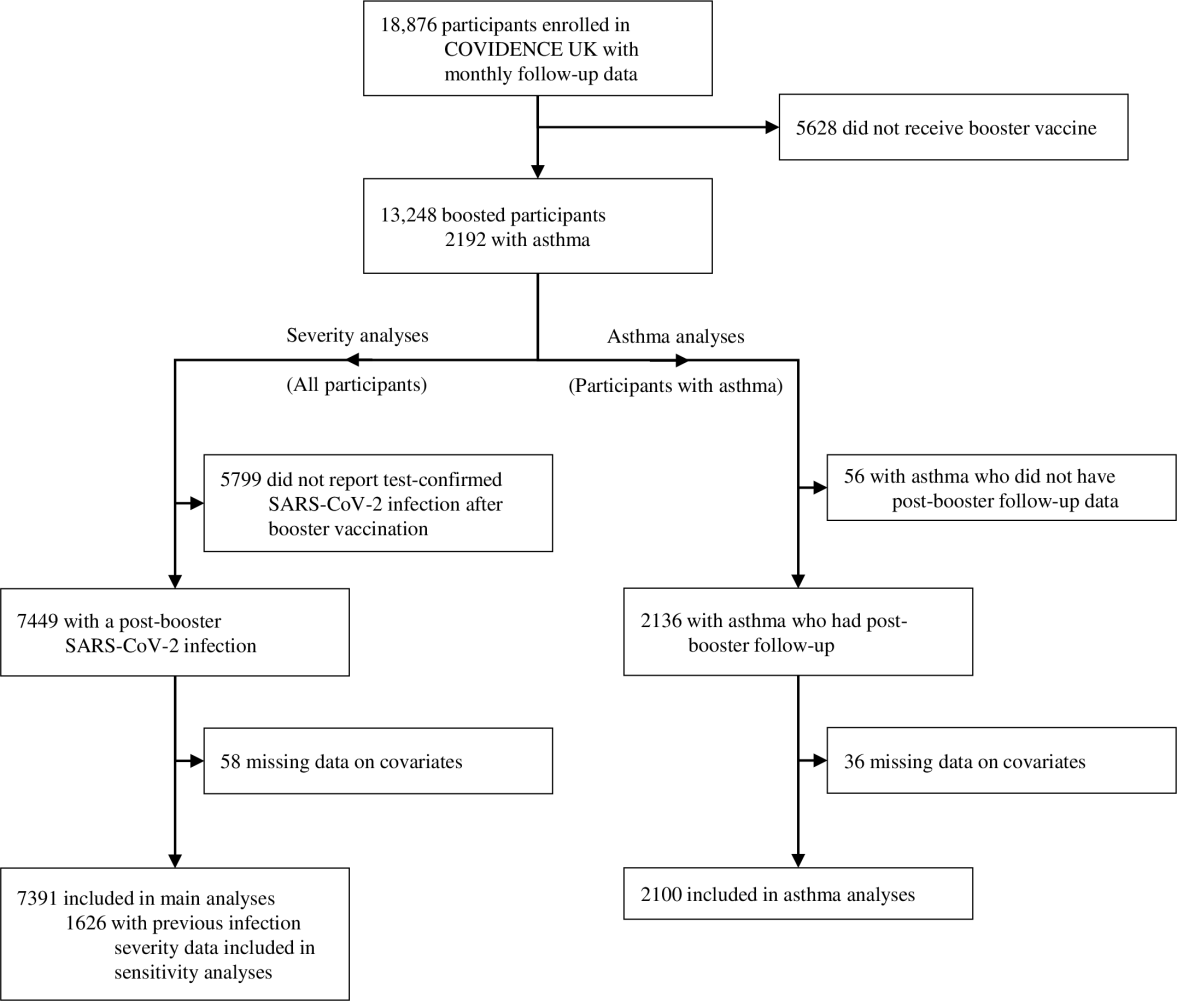
Participant flow diagram. Online supplemental table S2 presents a comparison of participant characteristics between boosted participants with and without reported breakthrough infections.

Between August 2021 and January 2024, 8689 SARS-CoV-2 infections were reported by 7391 boosted participants (figure 2; online supplemental figure S2 and table S2). 6286 (72.3%) of infections were recorded after access to free testing had ceased (online supplemental figure S3). Participants completed a median of 14 questionnaires (IQR 12–17) after their first booster vaccination; among the 1163 (15.7%) participants who missed at least one questionnaire, the median completion rate was 90% (81–94).

Participants had a median age of 63 years (IQR 55–69), 5368 (72.6%) were female and 7139 (96.6%) were white (table 1; online supplemental table S3). 2770 (31.9%) of 8689 post-booster infections led to participants reporting being very unwell: 1430 (27.5%) of 5205 infections occurring in participants vaccinated in the previous 6 months, 1097 (37.9%) of 2897 infections in participants vaccinated 6–12 months prior, and 243 (41.4%) of 587 infections occurring in participants vaccinated more than 1 year prior. The number of vaccines received before post-booster infection was slightly lower in participants reporting being very unwell (table 1). In this boosted population, more than 99% of infections occurred after the Omicron variant became dominant (17 December 2021;^6^ online supplemental figure S3) and fewer than 0.5% of infections led to hospitalisation.

**Table 1:** Participant characteristics, by severity of post-booster COVID-19

	Very unwell (N=2575)	Asymptomatic, mild or moderate (N=4816)	All participants (N=7391)
Sociodemographics			
Age, years	60 (51–67)	64 (57–70)	63 (55–69)
<50	556 (21.6%)	637 (13.2%)	1193 (16.1%)
50 to <65	1169 (45.4%)	1976 (41.0%)	3145 (42.6%)
65 to <75	724 (28.1%)	1823 (37.9%)	2547 (34.5%)
≥75	126 (4.9%)	380 (7.9%)	506 (6.8%)
Sex			
Female	2064 (80.2%)	3304 (68.6%)	5368 (72.6%)
Male	511 (19.8%)	1512 (31.4%)	2023 (27.4%)
Ethnicity			
Black, African, Caribbean, or Black British	4 (0.2%)	22 (0.5%)	26 (0.4%)
South Asian	32 (1.2%)	47 (1.0%)	79 (1.1%)
White	2472 (96.0%)	4667 (96.9%)	7139 (96.6%)
Mixed, multiple, or other ethnic groups	67 (2.6%)	80 (1.7%)	147 (2.0%)
Quartiles of IMD decile			
Q4 (least deprived)	863 (33.5%)	1703 (35.4%)	2566 (34.7%)
Q3	679 (26.4%)	1289 (26.8%)	1968 (26.6%)
Q2	511 (19.8%)	965 (20.0%)	1476 (20.0%)
Q1 (most deprived)	522 (20.3%)	859 (17.8%)	1381 (18.7%)
Highest educational level attained			
Primary or secondary	206 (8.0%)	472 (9.8%)	678 (9.2%)
Higher or further (A levels)	311 (12.1%)	680 (14.1%)	991 (13.4%)
College or university	1132 (44.0%)	2198 (45.6%)	3330 (45.1%)
Post-graduate	926 (36.0%)	1466 (30.4%)	2392 (32.4%)
Frontline health or care worker	272 (10.6%)	393 (8.2%)	665 (9.0%)
Booster vaccines received			
1	612 (23.8%)	980 (20.3%)	1592 (21.5%)
2	756 (29.4%)	1302 (27.0%)	2058 (27.8%)
≥3	1207 (46.9%)	2534 (52.6%)	3741 (50.6%)
Infection details			
Symptom duration, days*	21 (11–32)	12 (7–22)	14 (8–26)
Time since last vaccination, days*†	176 (104–270)	148 (89–232)	156 (94–245)
Vaccinated in past 6 months*	1661 (52.4%)	3544 (64.2%)	5205 (59.9%)
Vaccinated in past 12 months*	2877 (90.7%)	5225 (94.7%)	8102 (93.2%)
Any previous infections	190 (7.4%)	376 (7.8%)	566 (7.7%)
Previous infection severity			
Asymptomatic	20 (9.8%)	47 (11.6%)	67 (11.0%)
Mildly unwell	30 (14.7%)	113 (27.9%)	143 (23.5%)
Moderately unwell	46 (22.5%)	140 (34.6%)	186 (30.5%)
Very unwell	108 (52.9%)	105 (25.9%)	213 (35.0%)
Clinical characteristics			
BMI, kg/m^2^	25.2 (22.6–29.1)	24.9 (22.4–28.0)	25.0 (22.5–28.3)
<25	1239 (48.1%)	2492 (51.7%)	3731 (50.5%)
25 to <30	787 (30.6%)	1550 (32.2%)	2337 (31.6%)
≥30	549 (21.3%)	774 (16.1%)	1323 (17.9%)
Asthma	559 (21.7%)	712 (14.8%)	1271 (17.2%)
Diabetes	106 (4.1%)	203 (4.2%)	309 (4.2%)
Heart disease	95 (3.7%)	186 (3.9%)	281 (3.8%)
Kidney disease	506 (19.7%)	1101 (22.9%)	1607 (21.7%)
Hypertension	53 (2.1%)	91 (1.9%)	144 (1.9%)
Systemic immunosuppressants	167 (6.5%)	230 (4.8%)	397 (5.4%)
Oral corticosteroids	101 (3.9%)	119 (2.5%)	220 (3.0%)
Clinical risk group	730 (28.3%)	1155 (24.0%)	1885 (25.5%)
COPD or poorly controlled asthma	370 (14.4%)	449 (9.3%)	819 (11.1%)

Data are n (%), n/N (%) or median (IQR). Participants are classified as being very unwell if they reported requiring bedrest for at least one post-booster infection.

* 1206 participants reported more than one post-booster infection, so symptom duration and time since vaccination are given for 8689 included infections.

† The distribution of time since vaccination is shown in the appendix (Figure S4online supplemental figure S4).

BMI, body mass index; COPD, chronic obstructive pulmonary disease; IMD, Index of Multiple Deprivation.

1330 (18.0%) participants were eligible for 6-monthly vaccination and 5392 (73.0%) were initially eligible for annual vaccination; after the change in eligibility criteria for the autumn 2023 campaign, this number reduced to 3819 (51.7%), leaving 2242 (30.3%) participants ineligible for any further booster vaccinations. Approximately 65% of participants reported receiving all booster vaccinations they were eligible for.

Across all eligibility groups, greater time since vaccination was associated with increased odds of reporting being very unwell, although the small number of participants with respiratory disease compromised the statistical power of the analysis (table 2). Although point estimates consistently suggested that greater time since vaccination might be associated with a longer time to recovery, evidence for vaccination subgroups was weak (table 2). Less recent vaccination was associated with a small decrease in health-related quality of life overall and among those younger than 75 years (table 2); however, this did not reach any of our estimates for a minimum clinically important difference (online supplemental appendix p 17).

**Table 2:** Associations between time since vaccination and COVID-19 severity, speed of recovery, and health-related quality of life, by vaccine eligibility subgroup

	Severity: very unwell*		Speed of recovery		Change in EQ-5D index	
	OR (95% CI)	P value	HR (95% CI)	P value	Beta (95% CI)	P value
Association with not having received a booster vaccination in previous 12 months						
All participants	1.34 (1.11 to 1.62) (n=8689)	0.002	0.90 (0.81 to 0.99) (n=8659)	0.039	−0.02 (−0.03 to −0.00) (n=8153)	0.002
<65 years	1.31 (1.06 to 1.62) (n=5187)	0.013	0.94 (0.83 to 1.06) (n=5171)	0.291	−0.02 (−0.04 to −0.00) (n=4852)	0.013
Participants with controlled asthma†	1.62 (0.85 to 3.08) (n=635)	0.139	0.78 (0.55 to 1.11) (n=633)	0.168	0.00 (−0.05 to 0.05) (n=597)	0.995
Association with not having received a booster vaccination in previous 6 months						
All participants	1.56 (1.39 to 1.75) (n=8689)	<0.001	0.96 (0.90 to 1.01) (n=8659)	0.123	−0.01 (−0.02 to −0.00) (n=8153)	0.002
65 to <75 years	1.44 (1.17 to 1.76) (n=2939)	<0.001	0.95 (0.86 to 1.04) (n=2926)	0.238	−0.02 (−0.03 to −0.01) (n=2779)	0.005
Clinical risk group	1.61 (1.29 to 2.01) (n=2255)	<0.001	0.96 (0.86 to 1.08) (n=2250)	0.522	0.00 (−0.02 to 0.01) (n=2114)	0.628
Respiratory risk group‡	1.38 (1.00 to 1.92) (n=992)	0.054	0.94 (0.79 to 1.11) (n=989)	0.451	0.00 (−0.04 to 0.03) (n=917)	0.767

Table presents results for all participants and categorised by subgroups not eligible for annual boosters (<65 years, or with controlled asthma) or not eligible for 6-monthly boosters (aged 65 to <75 years, or in a clinical risk or respiratory risk group). All estimates are adjusted for age and other vaccine eligibility criteria, sex, ethnicity, socioeconomic status, general health, previous SARS CoV-2 infection, the number of vaccines received at time of infection, and free testing availability. Unadjusted results are presented in the appendix (Table S4online supplemental table S4). In all analyses, the reference group is participants who received a booster vaccine within the time frame indicated.

* Versus all other severities.

† Includes all participants with asthma, who have not reported at least two courses of oral corticosteroids in the preceding 24 months or at least one hospital admission for asthma in the preceding 24 months, and who are not on maintenance oral corticosteroids. Excludes participants with COPD.

‡ Includes participants with poorly controlled asthma or COPD.

COPD, chronic obstructive pulmonary disease.

Adjusting for previous infection severity attenuated some of our findings on symptom severity, most notably when considering 6-monthly vaccination; however, small sample sizes led to reduced statistical power, particularly for the clinical risk and respiratory subgroups (online supplemental table S5). Inverse probability-weighted regressions produced estimates that were largely similar to our main results (online supplemental table S6). Relaxing the assumption of independent censoring had no effect on our estimates for most subgroup analyses, with the largest variation shown for the respiratory risk group (HR varying from 0.99 (95% CI 0.83 to 1.18) for γ = –10 to 0.91 (0.77 to 1.07) for γ = 10; online supplemental figure S5), suggesting that the assumption of independent censoring had not been violated.

2100 participants with asthma were included in our exacerbation analysis (figure 2), of whom 1263 (60.1%) reported at least one SARS-CoV-2 infection between October 2021 and December 2023. The distribution of booster vaccinations, asthma exacerbations, and infections in this cohort is shown in online supplemental figure S2. Participants with greater average time between booster vaccinations were younger, more likely to be female, and less likely to be in a clinical risk group, and reported more SARS-CoV-2 infections and asthma exacerbations during follow-up (online supplemental table S7). Incident SARS-CoV-2 infection associated with increased risk of asthma exacerbation in boosted participants, both within a year of the last booster vaccination and later (table 3). For severe asthma exacerbations, point estimates were higher for infections occurring more than 1 year after the last vaccination, although CIs overlapped (table 3).

**Table 3:** Odds of asthma exacerbations after SARS-CoV-2 infection (vs no infection) by time since vaccination

	≤12 months	>12 months
Any exacerbation		
Unadjusted	2.87 (2.38 to 3.45)	3.35 (1.79 to 6.27)
Adjusted	5.11 (4.19 to 6.24)	5.60 (2.98 to 10.53)
Severe exacerbation		
Unadjusted	2.75 (2.02 to 3.74)	5.90 (2.29 to 15.23)
Adjusted	6.59 (4.70 to 9.22)	9.20 (3.56 to 23.78)

Data are odds ratio (95% CI). 2136 participants were included in unadjusted analyses and 2100 in adjusted analyses. Adjusted odds ratios are adjusted for age and other vaccine eligibility criteria, sex, ethnicity, socioeconomic status, general health, previous SARS CoV-2 infection, the number of vaccines received at time of infection, asthma treatment level, asthma exacerbation history, incident non-COVID-19 acute respiratory infection, tobacco smoking status, season, and measures of response rate, SARS-CoV-2 testing rate, and test availability. In all analyses, the reference group is participants who did not report a SARS-CoV-2 infection.

## Discussion

In this large, prospective, observational study of UK adults receiving booster vaccinations against SARS-CoV-2, we show that longer time since last vaccination was consistently associated with worse COVID-19 symptom severity, in all subgroups considered. However, while increased time since last vaccination was associated with a reduction in health-related quality of life after subsequent SARS-CoV-2 infection in participants younger than 75 years, the reduction was small and unlikely to be clinically significant. We found some evidence that more recent vaccination associates with shorter symptom duration, but evidence for vaccination subgroups was weak. When considering participants with asthma, incident SARS-CoV-2 infection continued to associate with asthma exacerbations in a boosted population. Importantly, our findings suggest that the risk of severe exacerbations may increase with greater time since booster vaccination, although we lacked the power to show a clear effect.

Previous studies have shown decreases in vaccine effectiveness in the first 6–8 months following a booster vaccination,^15 17 32^ accompanied by greater symptom severity when infected^12 13^ and increased risk of severe or critical COVID-19.^14^,^16^ However, few have considered symptom duration,^12^ and we are not aware of any that have looked at the effect of time since last booster vaccination on health-related quality of life. By considering these three elements of acute infection, we explore the impact of frequent booster vaccinations from multiple perspectives.

We observed a consistent relationship between self-reported symptom severity and time since vaccination; however, these results rarely translated to changes in symptom duration or health-related quality of life—and where they did, changes observed were unlikely to be clinically significant (online supplemental appendix p 17). Nonetheless, the impact of reducing the severity of the acute infection—particularly among working-age adults—should not be underestimated. While 28% of our participants vaccinated within the past 6 months required bed rest, this proportion increased to 41% of those who had not been vaccinated for more than a year. Given that more than a third of UK organisations surveyed reported COVID-19 to be among the top three causes of short-term sickness absence in their workforce,^33^ the differences in acute COVID-19 severity we observed have the potential to translate to a huge number of working days lost.

Eligibility criteria for SARS-CoV-2 booster vaccinations in the UK have been narrowed each autumn, meaning an increasing portion of the UK population has not been vaccinated for more than a year. Among this population are people with well-controlled asthma, who have not been eligible for a booster vaccine since autumn 2021. We found that incident SARS-CoV-2 infection remained associated with asthma exacerbations in boosted participants, independently of asthma severity and exacerbation history. While vaccination is known to reduce the severity of the acute infection, thus potentially reducing exacerbation risk,^34^ assessment of electronic health records has shown a greater percentage of asthmatic patients being prescribed systemic corticosteroids or antibiotics when infected with Omicron as opposed to earlier variants of SARS-CoV-2,^35^ suggesting that the Omicron variant may be triggering more asthma exacerbations. This is supported by paediatric studies, which have shown higher odds of asthma exacerbations with Omicron compared with other variants.^36^ Therefore, despite Omicron being associated with milder disease than previous variants,^37^ its impact on those at risk of asthma exacerbations remains elevated, emphasising the importance of vaccinating this population.

Although the difference was not significant, our point estimates suggest that people with asthma vaccinated more than 1 year ago may be at increased risk of severe exacerbations than those vaccinated within the past year, when infected with SARS-CoV-2. Further, well-powered studies are urgently needed to assess this point conclusively, particularly in people with well-controlled asthma who have not been recently vaccinated. In particular, the classification of asthma control should be made cautiously in these years following the acute pandemic,^38^ as lower exposure to viruses and greater adherence to preventive behaviours mean that recent exacerbation history may not be fully representative of someone’s risk of future exacerbations.^39 40^

This study has several strengths. By considering symptom severity and duration, health-related quality of life, and asthma exacerbations, we present a multifaceted picture of the acute impact of breakthrough SARS-CoV-2 infection. The large size of our dataset allowed us to look at outcomes in specific subgroups of the population, according to current vaccine eligibility. Extended follow-up meant we could assess the impact of annual vaccination, whereas previous studies have mostly focused on outcomes up to 6 months post vaccination,^12^,^1416^ and thus cannot accurately represent risk for populations no longer eligible for additional booster vaccinations. Additionally, our use of 6-month and 12-month thresholds, rather than shorter thresholds used in other studies,^12 13 32^ makes it easier to interpret our findings within the context of vaccination programmes offering boosters every 6–12 months.^2^ Monthly follow-up questionnaires enabled repeated-measures analysis, allowing us to control for time-varying covariates when considering exacerbation risk. Detailed information on both outcomes and exposures enabled us to minimise confounding with adjusted analyses.

This study also has some limitations. With very few hospitalisations, we were unable to assess the association between time since vaccination and severe disease; however, mild or moderate COVID-19 still has a substantial impact on society, with boosted individuals taking an average of 5 days’ sick leave to recover.^41^ By treating time as categorical rather than continuous, we will have lost some nuance on the effect of time since vaccination. However, our aim was to reflect the impact of existing vaccination policy, and we believe our approach has aided the interpretability of our results with respect to policy. We lacked power in some subgroup analyses, particularly for asthma exacerbations, meaning that further study in larger populations is still needed to confirm or refute the associations reported. We did not have data on all the eligibility criteria for the clinical risk group, potentially leading to some misclassification of vaccine eligibility, which may have attenuated some of our results. As our data are self-reported and collected monthly, there is likely to be some level of information bias. However, by adjusting for whether infections were recorded before or after the reduction in free COVID-19 testing, we have controlled for the fact that asymptomatic and mild infections were less likely to be recorded (although still made up 30% of infections reported). Additionally, by binarising time since vaccination, we will have reduced imprecisions in self-reported vaccination dates. The majority of infections were recorded after access to free testing had ceased, meaning that our results may not be representative for people with limited access to tests, such as those from lower socioeconomic status. Additionally, conditioning on the likelihood of taking a SARS-CoV-2 test may have put us at risk of collider bias. However, our inverse probability-weighted analyses, which incorporated various determinants of testing such as socioeconomic status, produced similar estimates to our main results, lending strength to our findings. COVIDENCE UK is a self-selected cohort, meaning that some groups—such as women, older people and those of white ethnicity—are over-represented in our study, potentially limiting the generalisability of our results. Finally, as an observational study, we cannot rule out the possibility of unmeasured or residual confounding influencing our findings. However, the richness of data in COVIDENCE UK, and use of directed acyclic graphs, means that we were able to control for a large number of potential confounders in our analyses.

In conclusion, we found that longer time since booster vaccination was consistently associated with worse symptom severity of breakthrough SARS-CoV-2 infections, but had little effect on the speed of recovery or acute changes to health-related quality of life. We also observed that incident COVID-19 continued to associate with increased risk of asthma exacerbations in a boosted population, independent of asthma severity and exacerbation history, and that risk of severe exacerbations may increase with longer time since last vaccination. These findings highlight the importance of continuing to vaccinate those who fulfil existing eligibility criteria for boosters, particularly given widespread under-vaccination,^9^ and the urgent need for further research on the impacts of vaccination in people with asthma who are no longer eligible for booster vaccinations.

## Supplementary material

10.1136/bmjresp-2025-003158 online supplemental file 1

## Data Availability

Data are available upon reasonable request.
